# Efficacy on radiofrequency ablation according to the types of benign thyroid nodules

**DOI:** 10.1038/s41598-021-01593-9

**Published:** 2021-11-15

**Authors:** Jianhua Li, Wenping Xue, Pengfei Xu, Zhen Deng, Caiwen Duan, Danhua Zhang, Shouhua Zheng, Kefei Cui, Xinguang Qiu

**Affiliations:** 1 Department of Thyroid Surgery, The First Affiliated Hospital of Zhengzhou University, No. 1 Jianshe East Road, Zhengzhou, 450052 Henan People’s Republic of China; 2 Department of Ultrasound, The First Affiliated Hospital of Zhengzhou University, No. 1 Jianshe East Road, Zhengzhou, 450052 Henan People’s Republic of China

**Keywords:** Endocrine system and metabolic diseases, Clinical trials, Outcomes research

## Abstract

Percutaneous radiofrequency ablation (RFA) has been recommended as minimally invasive treatment for patients with symptomatic benign thyroid nodules (BTNs) because of the large number of clinical applications. This retrospective observational study sought to evaluate the clinical outcomes of RFA for BTNs. From 2014 to 2019, a sample size of 1289 patients treated by RFA were 262 ones with solid nodules and 1027 ones with cystic-solid nodule, respectively. The efficacy including the nodule maximal diameter reduction ratio (MDRR), the volume reduction ratio (VRR) and the cosmetic scores reduction ratio (CSRR). The results of the nodule MDRR and VRR in the cystic-solid nodule group were significantly better than those in the solid nodule group at the 3rd and 6th month, and the CSRR in the two groups showed statistically significant difference at the 3rd month. In a word, RFA is an effective method for symptomatic benign solid or cystic-solid nodules. The achieved MDRR and VRR in the cystic-solid nodule group were significantly better than those in the solid nodule group at the 3rd and 6th month.

## Introduction

Thyroid nodules are a common finding in general population. They can be approximatively discovered in 20–76% by ultrasound (US) scan, and in 3–7% by palpation^1^. Most nodules are asymptomatic and benign, and can be managed by observation only. However, even benign nodules may cause problems, such as symptoms of tracheal and esophageal compression, secondary to or combined with hyperthyroidism, retrosternal goiter, cosmetic concerns and psychological burdens^2^. The efficacy of drug therapy for thyroid nodules is not obvious, surgery and radioactive iodine therapy are still considered the mainstay of treatment, but both of these options have drawbacks. Radioiodine therapy is mainly aimed at the benign nodules of thyroid adenoma with hyperthyroidism, however, it is easy to be associated with hypothyroidism. In particular, surgery carries a 2–10% complication rate and requires general anesthesia and hospitalization^3^. Furthermore, it is expensive, and may cause problems such as scar formation and iatrogenic hypothyroidism. Ultrasound-guided minimally invasive treatment has the advantages of less trauma, less complications, no scar on the neck, short treatment time, safe, effective, repeatable treatment etc., and is generally used in clinic. Minimally invasive therapy is mainly divided into chemical ablation and thermal ablation. Thermal ablation refers to radiofrequency ablation (RFA), microwave ablation (MWA), laser ablation (LA) and high-intensity focused ultrasound (HIFU)^4–7^.

Some studies^8,9^ explored the possibility of a combination of radioiodine and thermal ablation for the treatment of benign thyroid diseases. The combined therapy significantly decreases the required dose of ^131^I and hospitalization time. However, this method is still in the experimental stage with short follow-up and limited patients. Its safety and efficacy need to be evaluated by further large data.

This study applied the moving shot technique for monopolar RFA. With this method, a large thyroid nodule can be successfully ablated. Both monopolar and bipolar RFA have their own superiorities and drawbacks. In contrast to monopolar RFA, bipolar RFA works more comfortably and saves time with the reason that a grounding pad is not needed to be placed^10^. Additionally, the required grounding pad for monopolar RFA could induce skin burns and increase the risk of breakdown of implanted cardiac pacemakers^11^. Even though monopolar RFA has its own theoretical drawback, the promising bipolar RFA still does not be applied in clinic now. The safety and efficacy of bipolar RFA have not been thoroughly proved due to the small case number just like the combined therapy mentioned above.

Ultrasound-guided RFA has exhibited distinct efficacy for benign thyroid nodules via decreasing the nodular volume by 84.8%^3,12^. At present, the indications of radiofrequency ablation are gradually increasing, including parathyroid hyperplasia, papillary thyroid microcarcinoma and metastatic lymph nodes. Thermal ablation is considered a better alternative therapy for single papillary thyroid carcinoma without capsule invasion or lateral cervical metastasis. Although surgical resection is the first choice for patients with recurrent thyroid cancer, if the patients have severe scars and fibroplasia in the operation area and cannot tolerate or refuse second surgery, radiofrequency ablation of the cancer focus and related lymph nodes can be performed. According to a recent meta-analysis of RFA for locally recurrent thyroid cancer, the curative success is 100%, with a serum thyroglobulin decrease of 71.6%^13^. This retrospective observational study compared the clinical outcomes after a single RFA of solid nodules vs. cystic-solid nodules in a mass of patients with benign lesions.

## Materials and methods

### Patients

**Figure 1:**
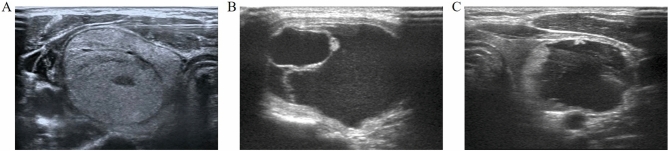
Different types of benign thyroid nodules (BTNs). (**A**) US image of a solid thyroid nodule. (**B**) US image of a cystic thyroid nodule. (**C**) US image of a colloid thyroid nodule.

**Figure 2:**
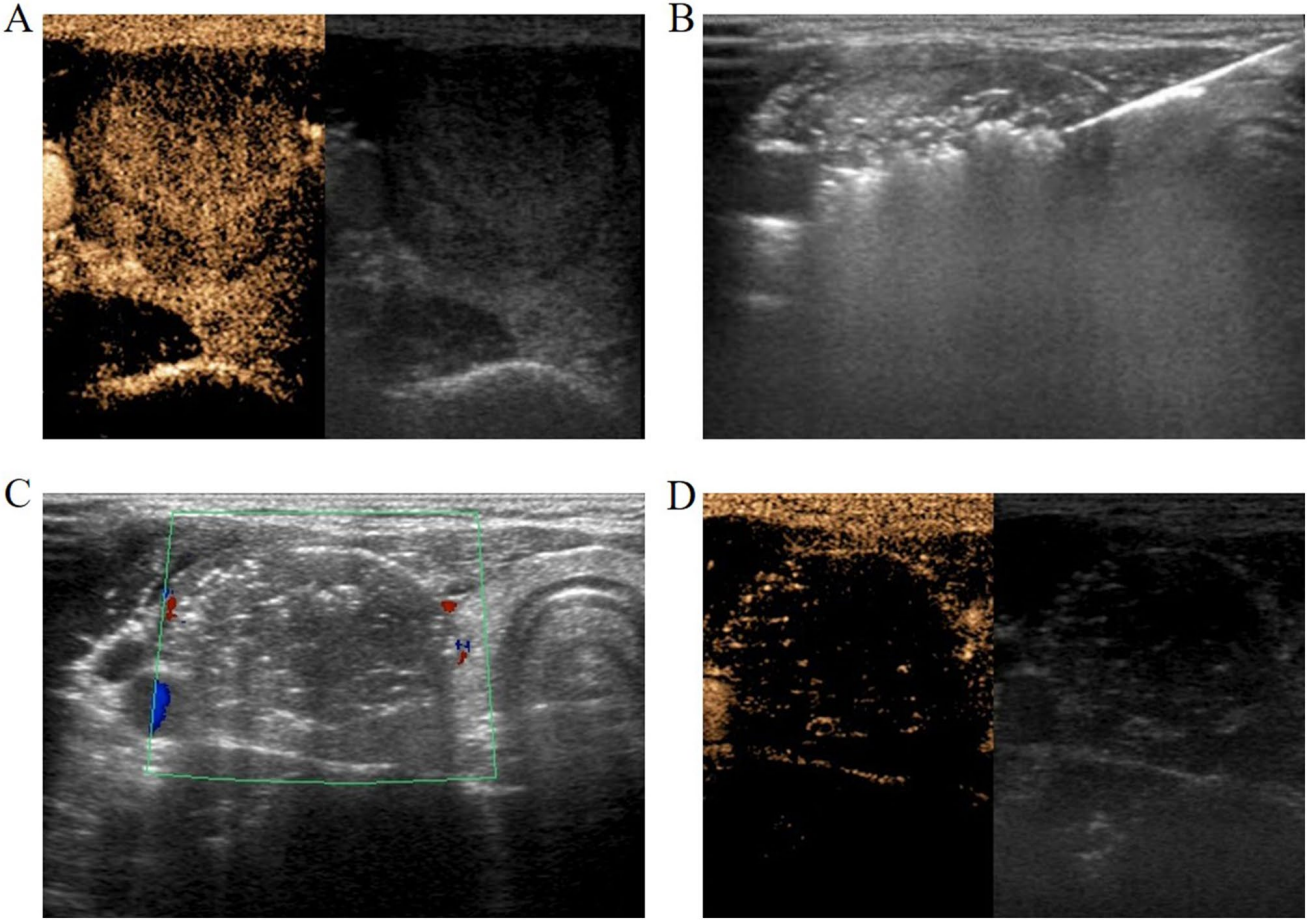
RFA procedure of a benign thyroid nodule. (**A**) Preoperative contrast enhanced ultrasound (CEUS) was used to define the blood perfusion of the nodule and evaluate the benign signs. (**B**) Ablation was performed layer-by-layer using the moving-shot method: the needle inside the lesion with the appearance of a hyperechogenic area represented the ablated area. (**C**) Gland was basically ablated. Most of the glandular region is coated with coagulated and necrotic hyperechoic vaporization region. (**D**) Postoperative contrast enhanced ultrasound (CEUS) was applied to assess the thoroughness of ablation.

**Figure 3:**
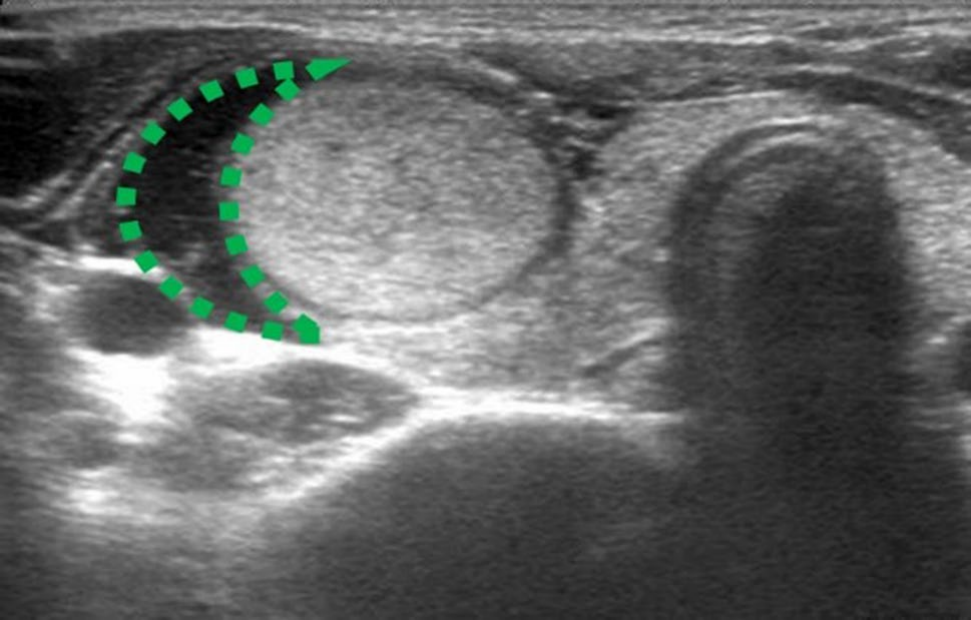
The hydrodissection with continuous fluid infusion. A buffering zone (green dotted line) was created to establish a liquid isolation zone > 0.5 cm in depth between the thyroid and adjacent structures.

This study was approved by the ethical and scientific review board of the First Affiliated Hospital of Zhengzhou University (2019-KY-264). Written informed consent was obtained from all patients enrolled in this study. We confirm that all methods were performed in accordance with the relevant guidelines and regulations. From May 2014 to September 2019, we retrospectively studied 1289 patients (198 men, 1091 women) who were diagnosed as benign thyroid nodules (BTNs) by fine-needle aspiration biopsy (FNAB). They were alternately assigned into 2 groups according to the composition of BTNs: solid group (n = 262) and cystic-solid group (n = 1027). The common thyroid nodular types were: (1) Solid: solid portion more than 80% (Fig. 1A); (2) Cystic: cystic portion more than 80% (Fig. 1B); (3) Mixed (cystic-solid): not meeting the criteria for solid or cystic^14^.

The inclusion criteria were: (1) maximal diameter no less than 2 cm, progressive growth or enlargement more than 20% of the volume in the last year; (2) symptomatic nodules: compressive symptoms, neck discomfort, foreign body sensation, throat irritation, dyspnea; (3) cosmetic concerns; (4) clinical thyrotoxicosis and hyperthyroidism caused by autonomously functioning thyroid nodules (AFTNs); (5) refusal or contraindications to surgery. The exclusion criteria were: (1) severe cardiopulmonary dysfunction; (2) malignant or follicular thyroid neoplasms; (3) coagulation disorders; (4) vocal cord palsy in the contralateral side; (5) colloid nodules (Fig. 1C); (6) retrosternal goiter.

### Equipment

US-guided RFA was performed using grey-scale imaging with a iU22 US scanner and a high-frequency linear probe (L12-5) (Philips, The Netherlands), while contrast-enhanced ultrasound (CEUS) with a high-frequency linear probe (L9-3) was used to monitor the RFA procedures (Fig. 2A,D), as well as the initial diagnostic evaluation and the follow-up^15^. All patients were studied with US before the treatment by one of two radiologists (Kefei Cui and Yuanjing Huang) enrolled in the study, with 35 and 20 years of thyroid US experience, respectively.

### Pre-ablation assessment

Examination of the bilateral vocal cords with a laryngo-fiberscope and blood coagulation tests were performed before RFA and the values were within normal range in all patients. The cosmetic score^14,16^ was evaluated by an experienced clinician as follows: (1) no palpable mass; (2) no cosmetic problem but palpable mass; (3) a cosmetic problem on swallowing only; (4) easily visible mass.

### Image and clinical analysis

All medical records and US images were reviewed by experienced radiologists. Cosmetic and symptomatic problems of the patients and location, largest diameter, volume and the proportion of the solid component of each nodule were evaluated before and after RFA. For each thyroid nodule, the pre- and post-treatment volumes^14,16^ were calculated as V = πabc/6 (where V is the volume, a is the largest diameter, b and c are the two other perpendicular diameter).

### RFA procedure

The RFA system of the VIVA RF generator (VIVA RF generator, STARmed, Goyang, Korea) was applied in the study. An 18-guage, monopolar, modified, internal-cooled RFA antenna (VIVA, STARmed, Goyang, Korea) with a 1-cm active tip and a 7-cm shaft length was applied, which was specifically modified for the ablation of thyroid nodules^16^.

Patients were put in a supine position with hyperextended neck. Two ground pads were positioned on the patient’s thighs before the procedure. We used trans-isthmic and moving-shot techniques^17^. All procedures were completed under a sterile operation and local anesthesia with 2% lidocaine. An electrode was inserted in the same position of the local anesthesia site under US guidance, with the electrode tip initially positioned in the deepest and remote region of the lesion (Fig. 2B). The “moving shot technique” consists in the introduction of an internally cooled needle with variable diameter (17–19G), length (7–15 cm) and active tips (ranging from 0.5 to 2 cm). The needle is inserted through the isthmus (trans-isthmic approach), starting from the middle to the lateral direction, to reach the nodule which is divided in different small hypothetical zones, each ablated by moving the tip of the needle from the deepest position upwards to the most superficial part of the nodule. Finally, the ablation of the lesion is confirmed by the US appearance of a hyperechogenic area associated to a sudden increase of impedance registered by the external generator (Fig. 2C).

For mixed or mainly cystic nodules, fluid was aspirated under US-guided with a 18 gauge needle just before ablation. When the nodule was located in the upper or lower pole of the thyroid or adjacent to vital structures such as the vagus nerve, trachea or esophagus, hydrodissection^18^, consisting of 5% dextrose solution injection between the peripheral nodule area and surrounding critical structure, is aimed at avoiding thermal injury of adjacent structures (Fig. 3).

### Post-ablation assessment

The US presentation of ablated nodules, such as sizes, volumes, echogenicity, cosmetic scores of patients were evaluated and recorded at 3-, 6- and 12-month. The clinical efficiency of solid nodules and cystic-solid nodules were evaluated by comparing MDRR, VRR and CSRR. The maximal diameter reduction ratio (MDRR), volume reduction ratio (VRR) and cosmetic scores reduction ratio (CSRR) were calculated from the following formulations: (1) maximal diameter reduction ratio (%) = [100 (initial maximal diameter − final maximal diameter)]/initial maximal diameter; (2) volume reduction ratio (%) = [100 (initial volume − final volume)]/initial volume; (3) cosmetic scores reduction ratio (%) = [100 (initial cosmetic scores − final cosmetic scores)]/initial cosmetic scores.

### Statistical analysis

Data analysis was performed using the SPSS software (SPSS for windows 21.0, SPSS, Chicago, IL) and GraphPad Prism version 5.0 (California, USA). Continuous variables were as the mean ± standard deviation (SD). One-way ANOVA and the Student’s *t* test were used to analyze MDRR, VRR and CSRR. The χ^2^ test was applied for categorical variables. A value of p < 0.05 was considered to define statistically significant difference.

## Results

From May 2014 to September 2019, 1289 patients (198 men, 1091 women) with BTNs were admitted in the First Affiliated Hospital of Zhengzhou University according to the inclusion criteria and underwent RFA procedures. All patients’ thyroid functions including TSH, FT_3_ and FT_4_ were at normal ranges before ablation. The baseline characteristics of the BTNs in the two groups before RFA are presented in Table 1. For solid group patients, the mean maximal diameter, volume and cosmetic scores of nodules before RFA were 2.88 ± 1.39 cm, 5.60 ± 4.52 ml, 2.35 ± 0.97. For cystic-solid group patients, the mean maximal diameter, volume and cosmetic scores of nodules before RFA were 3.19 ± 1.47 cm, 8.12 ± 6.32 ml, 2.58 ± 0.94. Comparison of these three variables was different among the two groups (p < 0.05). In addition, there were no significant differences in the gender, age and follow-up time (p > 0.05). Based on the differences among base-lines, we only chose MDRR, VRR and CSRR to analyze the two groups, while for solid nodules or cystic-solid nodules themselves, we supplemented the analysis with data and reduction rate differences at the 3rd, 6th, 12th month.

**Table 1:** Clinical features of solid nodule group and cystic-solid nodule group patients.

Characteristics	Solid nodule group	Cystic-solid nodule group	p value
Gender (M/F) (n)	36/226	162/865	0.472
Age (years)	45.39 ± 14.52	45.26 ± 13.52	0.895
Maximal diameter (cm)	2.88 ± 1.39	3.19 ± 1.47	0.002
Volume (ml)	5.60 ± 4.52	8.12 ± 6.32	0.000
Cosmetic score	2.35 ± 0.97	2.58 ± 0.94	0.000
Follow-up (months)	5.05 ± 5.29	5.62 ± 6.88	0.286

Means ± SD are shown. p < 0.05 was considered to indicate statistically significant difference. *M/F* male/female, *cm* centimeter, *ml* milliliter.

**Figure 4:**
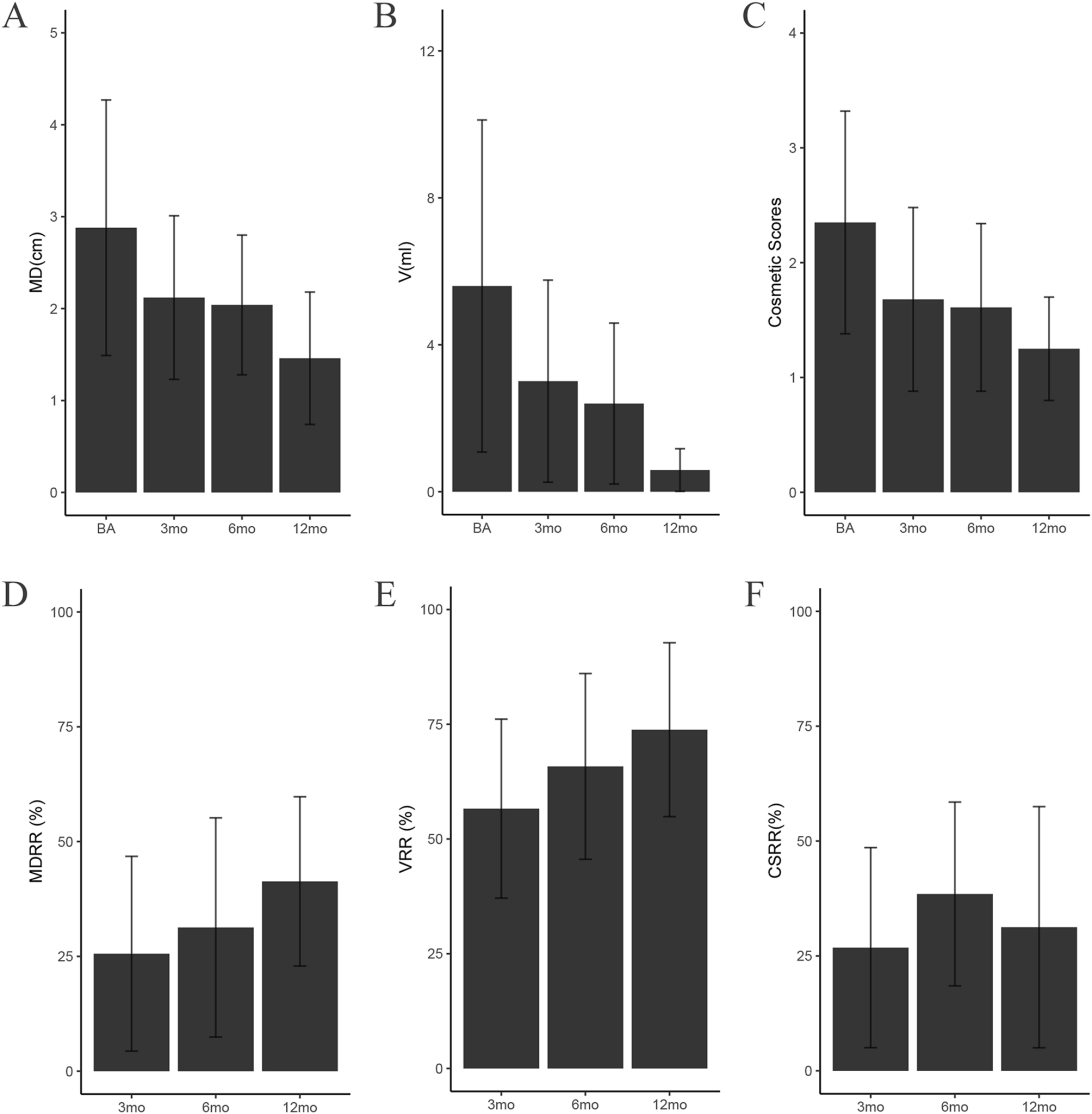
The comparison of the ablated nodular MD/V/CS/MDRR/VRR/CSRR in the solid nodule group at each follow-up. (**A**) The comparison of maximal diameter in the S_ group. (**B**) The comparison of volume in the S_ group. (**C**) The comparison of cosmetic scores in the S_ group. (**D**) The comparison of maximal diameter reduction ratio in the S_ group. (**E**) The comparison of volume reduction ratio in the S_ group. (**F**) The comparison of cosmetic scores reduction ratio in the S_ group. Means ± SD are illustrated. p < 0.05 was considered to indicate statistically significant difference (*MD* maximal diameter, *V* volume, *CS* cosmetic scores, *MDRR* maximal diameter reduction ratio, *VRR* volume reduction ratio, *CSRR* cosmetic scores reduction ratio, *S_* solid nodule group, *BA* baseline, *mo* months).

In the solid group, the mean maximal diameters of nodules at the 3rd, 6th, 12th month were 2.12 ± 0.89 cm, 2.04 ± 0.76 cm, 1.46 ± 0.72 cm, respectively, all of which were significantly less than that before RFA (p < 0.001) (Fig. 4A); the volume of nodules at the 3rd, 6th, 12th month were 3.01 ± 2.75 ml, 2.40 ± 2.19 ml, 0.59 ± 0.58 ml, respectively, all of which were significantly less than that before RFA (p < 0.001) (Fig. 4B); the cosmetic scores of nodules at the 3rd, 6th, 12th month were 1.68 ± 0.80, 1.61 ± 0.73, 1.25 ± 0.45, respectively, all of which were significantly less than that before RFA (p < 0.001) (Fig. 4C). Through further pairwise comparison, we found that there were no significant difference between 6 mo vs. 3 mo and 12 mo vs. 6 mo after RFA (p > 0.05). While 12 mo vs. 3 mo showed significant difference after RFA (p < 0.05), indicating that the solid nodule group shrank markedly at 12 mo after operation.

To further verify above results, we selected MDRR, VRR and CSRR associated with nodular maximal diameter (MD), volume (V) and cosmetic score (CS) as study subjects. The same differences could be observed in MDRR (Fig. 4D) and VRR (Fig. 4E). However, CSRR changed significantly at 6 mo, which may be explained by the variation of interior and exterior. The calculated maximum diameter and volume of solid nodules decreased after ablation over time. However, the cosmetic score is an external performance, even though the nodules are still shrinking at 12 mo, the appearance has been improved greatly at 6 mo in advance. Consequently, we could find that there were no significant differences between 3 mo vs. 12 mo and 6 mo vs. 12 mo after RFA (p > 0.05). Nevertheless, 6 mo vs. 3 mo showed significant difference after RFA (p < 0.05), the cosmetic scores were almost consistent at 6 mo and 12 mo (Fig. 4F). Taken together, these results demonstrated that the maximum diameter and volume of solid nodules group would become smaller and smaller with the extension of time, both shrank prominently at 12 mo during the observation period.

**Figure 5:**
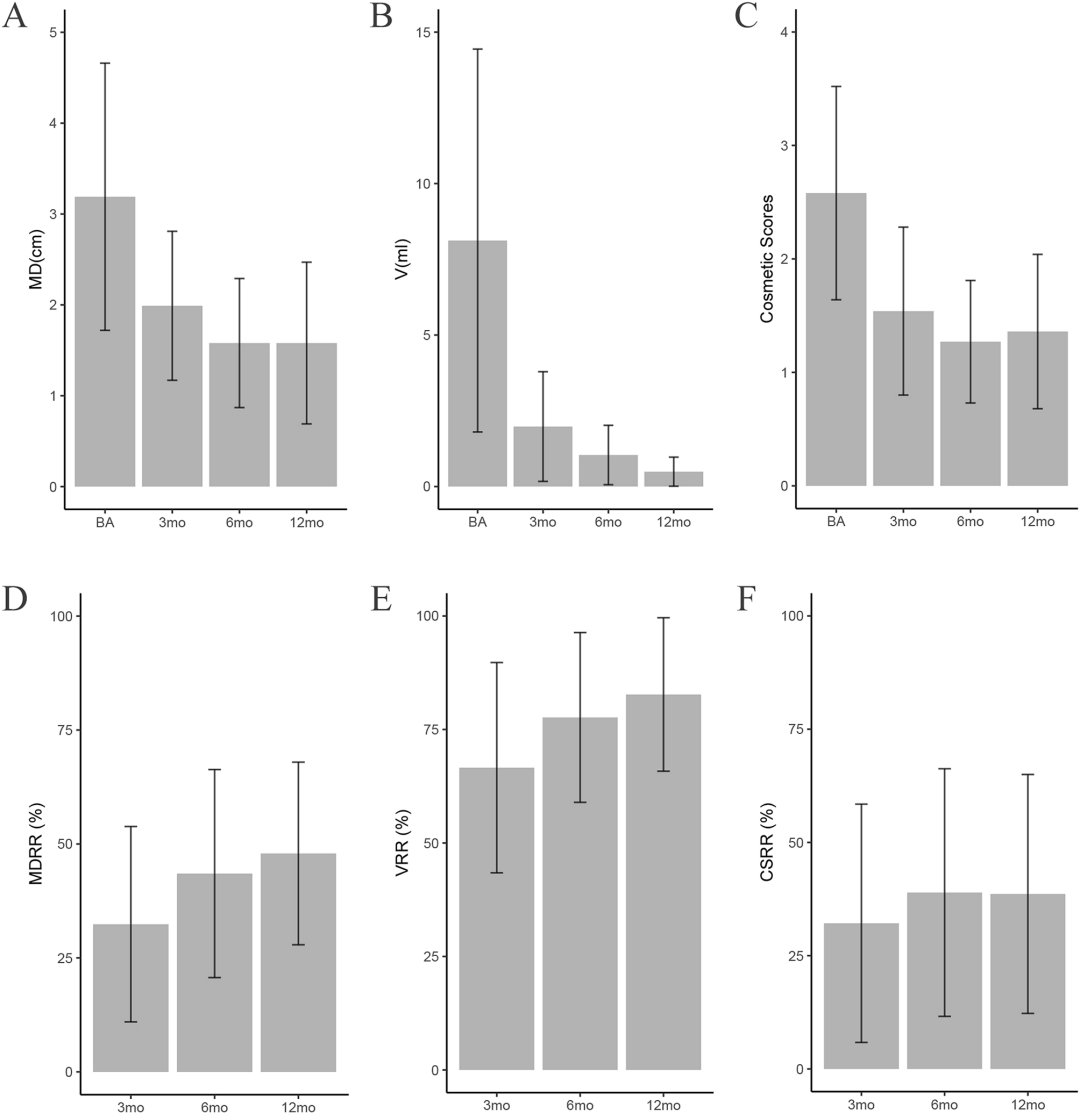
The comparison of the ablated nodular MD/V/CS/MDRR/VRR/CSRR in the cystic-solid nodule group at each follow-up. (**A**) The comparison of maximal diameter in the C_ group. (**B**) The comparison of volume in the C_ group. (**C**) The comparison of cosmetic scores in the C_ group. (**D**) The comparison of maximal diameter reduction ratio in the C_ group. (**E**) The comparison of volume reduction ratio in the C_ group. (**F**) The comparison of cosmetic scores reduction ratio in the C_ group. Means ± SD are presented. p < 0.05 was considered to indicate statistically significant difference (*MD* maximal diameter, *V* volume, *CS* cosmetic scores, *MDRR* maximal diameter reduction ratio, *VRR* volume reduction ratio, *CSRR* cosmetic scores reduction ratio, *C_* cystic-solid nodule group, *BA* baseline, *mo* months).

In the cystic-solid group, the mean maximal diameters of nodules at the 3rd, 6th, 12th month were 1.99 ± 0.82 cm, 1.58 ± 0.71 cm, 1.58 ± 0.89 cm, respectively, all of which were significantly less than that before RFA (p < 0.001) (Fig. 5A); the volume of nodules at the 3rd, 6th, 12th month were 1.98 ± 1.81 ml, 1.04 ± 0.98 ml, 0.49 ± 0.48 ml, respectively, all of which were significantly less than that before RFA (p < 0.001) (Fig. 5B); the cosmetic scores of nodules at the 3rd, 6th, 12th month were 1.54 ± 0.74, 1.27 ± 0.54, 1.36 ± 0.68, respectively, all of which were significantly less than that before RFA (p < 0.001) (Fig. 5C).

Some studies have found that larger MDRR and VRR can be achieved in the RFA group at 6 months^19^. Interestingly, similar results were observed in cystic-solid nodules group. By further analysis, we found that the change of MDRR (Fig. 5D) and VRR (Fig. 5E) was consistent to CSRR (Fig. 5F), all decreased obviously at 6 mo. The reason why these three variables showed synchronously is due to the different ablation method of cystic-solid nodules. The cystic fluid was aspirated before ablation, which may artificially accelerate the process of nodular shrinkage. Hence, the decline peak of nodular maximum diameter and volume appears in advance, synchronized with the cosmetic score. These results suggest that cystic-solid nodules shrank obviously at 6 mo after RFA.

**Figure 6:**
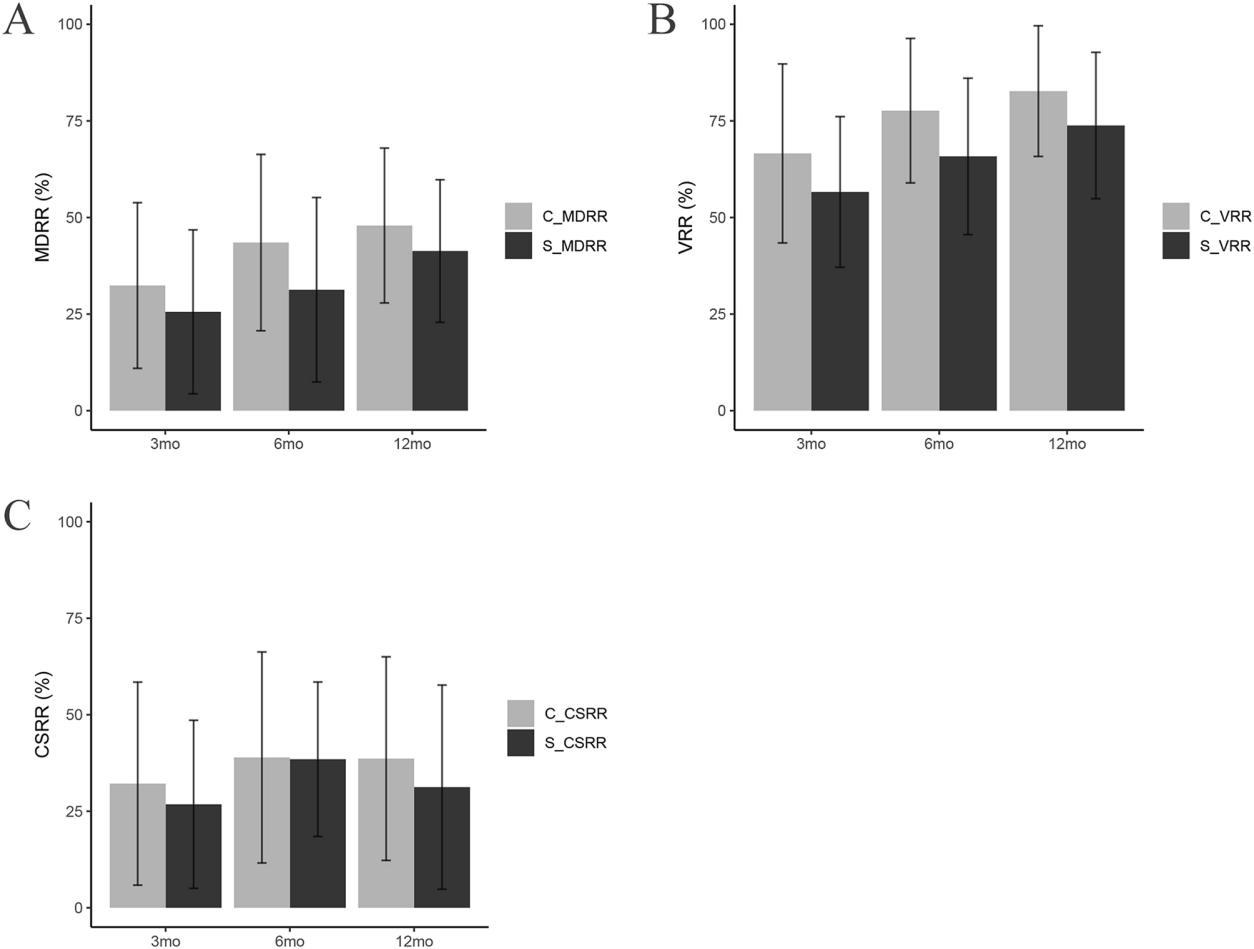
The comparison of the ablated nodular MDRR/VRR/CSRR between the solid and cystic-solid nodule group at each follow-up. (**A**) The comparison of maximal diameter reduction ratio between the two groups. (**B**) The comparison of volume reduction ratio between the two groups. (**C**) The comparison of cosmetic scores reduction ratio between the two groups. Means ± SD are demonstrated. p < 0.05 was considered to indicate statistically significant difference (*MDRR* maximal diameter reduction ratio, *VRR* volume reduction ratio, *CSRR* cosmetic scores reduction ratio, *S*_ solid nodule group, *C*_ cystic-solid nodule group, *BA* baseline, *mo* months).

In order to further compare the efficiency of the two groups after ablation, MDRR, VRR and CSRR were also selected as study subjects. The mean MDRRs of the solid group vs. the cystic-solid group at the 3rd, 6th, 12th month were 25.58 ± 21.21% vs. 32.40 ± 21.43% (p = 0.003), 31.30 ± 23.87% vs. 43.51 ± 22.82% (p < 0.010), 41.33 ± 18.44% vs. 47.92 ± 20.04% (p = 0.229), respectively (Fig. 6A).The mean VRRs of the two groups at the 3rd, 6th, 12th month were 56.61 ± 19.50% vs. 66.58 ± 23.16% (p < 0.001), 65.81 ± 20.24% vs. 77.65 ± 18.70% (p = 0.005), 73.81 ± 18.94% vs. 82.71 ± 16.90% (p = 0.106), respectively (Fig. 6B).The mean CSRRs of the two groups at the 3rd, 6th, 12th month were 26.80 ± 21.77% vs. 32.16 ± 26.30% (p = 0.031), 38.48 ± 20.00% vs. 38.94 ± 27.33% (p = 0.915), 31.25 ± 26.44% vs. 38.64 ± 26.37% (p = 0.335), respectively (Fig. 6C).

The mean nodular MDRRs and VRRs at the 3rd, 6th months showed significant difference between the two groups, but at the 12th month, statistical significance was not found, and the cystic-solid group had a higher MDRR and VRR than that in the solid group. Between the two groups, there was no significant difference in the mean CSRR at the 6th, 12th month. While at the 3rd month of the follow-up, statistical significance was found, and the cystic-solid group had a higher CSRR than that in the solid group.

Those results above may be attributed to different ablation method and transformation of nodular nature. The aspiration of cystic fluid could artificially increase the reduction ratio of cystic-solid nodules, which was higher than that of the solid nodules. Six months after ablation, cystic-solid nodules can completely convert into solid nodules, giving rise to no significant difference at 12 mo. Therefore, the 6 mo can be regarded as the confluent point of cystic-solid and solid nodules. Previously, the decrease rate of the two groups was different from each other, owing to their diverse composition. While after 6 months, the two groups performed synchronous rate of change due to the shift from cystic-solid to solid, and we can see that the difference was not significant at 12 mo.

**Figure 7:**
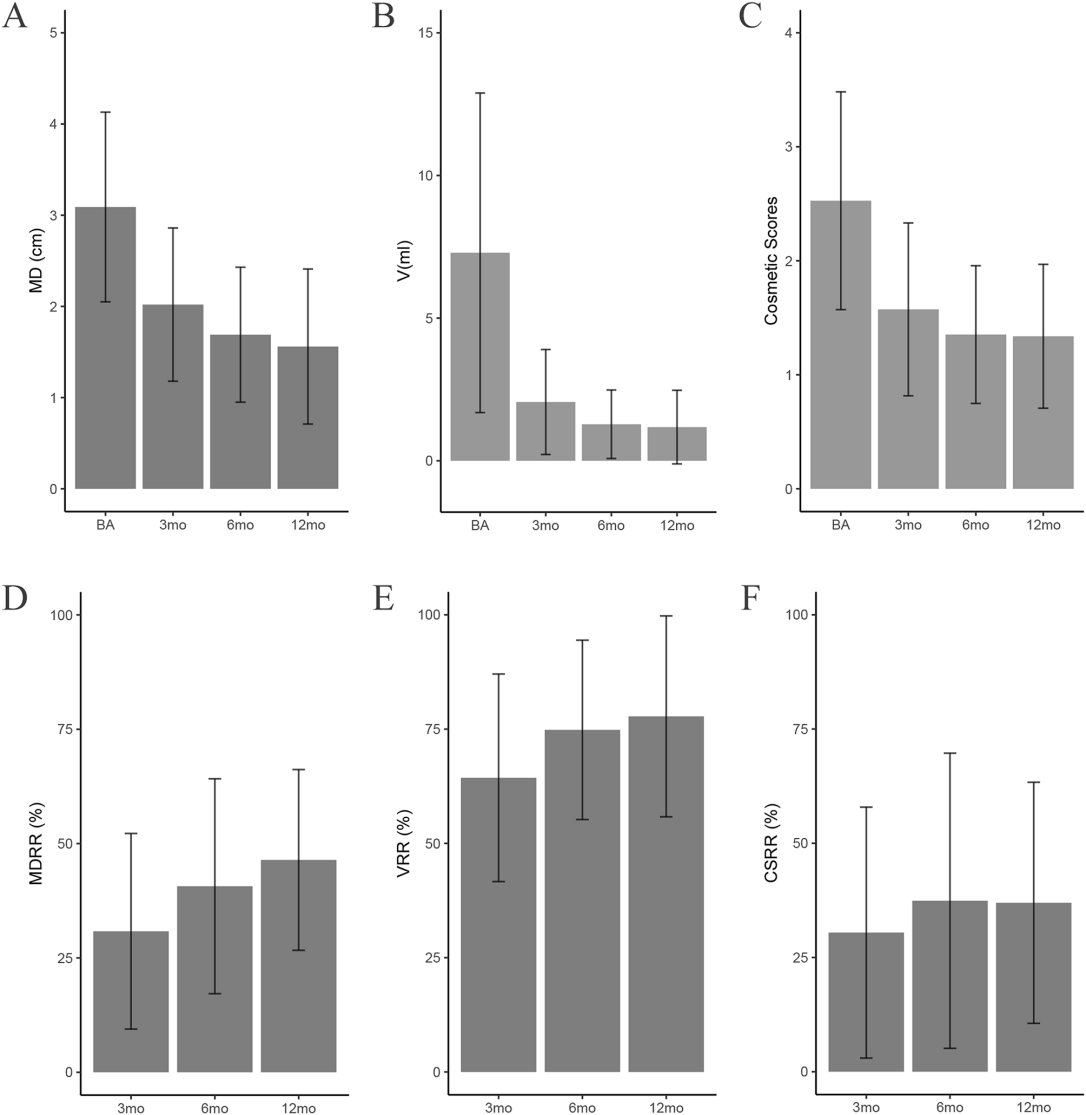
The comparison of the ablated nodular MD/V/CS/MDRR/VRR/CSRR in the total benign thyroid nodules (BTNs) group at each follow-up. (**A**) The comparison of maximal diameter in the BTNs_ group. (**B**) The comparison of volume in the BTNs_ group. (**C**) The comparison of cosmetic scores in the BTNs_ group. (**D**) The comparison of maximal diameter reduction ratio in the BTNs_ group. (**E**) The comparison of volume reduction ratio in the BTNs_ group. (**F**) The comparison of cosmetic scores reduction ratio in the BTNs_ group. Means ± SD are exhibited. p < 0.05 was considered to indicate statistically significant difference (*MD* maximal diameter, *V* volume, *CS* cosmetic scores, *MDRR* maximal diameter reduction ratio, *VRR* volume reduction ratio, *CSRR* cosmetic scores reduction ratio, *BTNs*_ benign thyroid nodules group, *BA* baseline, *mo* months).

The whole set of data was applied to testify that BTNs decreased most significantly at the 6th month after RFA^19^. Six comparative indices (MD, V, C, MDRR, VRR and CSRR) were selected for analysis. In the whole benign thyroid nodules group, the mean maximal diameter, volume and cosmetic scores of nodules before RFA were 3.09 ± 1.04 cm, 7.29 ± 5.60 ml, 2.53 ± 0.96; the mean maximal diameters of nodules at the 3rd, 6th, 12th month were 2.02 ± 0.84 cm, 1.69 ± 0.74 cm, 1.56 ± 0.85 cm, respectively (Fig. 7A); the volume of nodules at the 3rd, 6th, 12th month were 2.06 ± 1.84 ml, 1.28 ± 1.20 ml, 1.18 ± 1.29 ml, respectively (Fig. 7B); the cosmetic scores of nodules at the 3rd, 6th, 12th month were 1.57 ± 0.76, 1.35 ± 0.60, 1.34 ± 0.63, respectively (Fig. 7C). The mean MDRRs of BTNs at the 3rd, 6th, 12th month were 30.83 ± 21.38%, 40.68 ± 23.50%, 46.43 ± 19.76%, respectively (Fig. 7D). The mean VRRs of BTNs at the 3rd, 6th, 12th month were 64.35 ± 22.70%, 74.83 ± 19.62%, 77.79 ± 21.98%, respectively (Fig. 7E). The mean CSRRs of BTNs at the 3rd, 6th, 12th month were 30.45 ± 27.46%, 37.42 ± 32.30%, 36.97 ± 26.38%, respectively (Fig. 7F). All the above comparative indices before ablation vs. the observation time in the BTNs group significantly decreased; the p values were less than 0.001.

By pairwise comparison analysis, we found that there was statistical significance between 6 mo vs. 3 mo after RFA (p < 0.05), while 12 mo vs. 6 mo had no significant difference after RFA (p > 0.05), indicating that the BTNs shrank obviously at 6 mo after operation. Comparing solid nodules, cystic-solid nodules and all benign thyroid nodules, we found that during the 1-year follow-up period, solid nodules shrank most significantly at 12 mo. Whereas, cystic-solid nodules and benign thyroid nodules shrank most significantly at 6 mo, which is in line with most studies’ results. A recent systematic review and meta-analysis^20^ concluded that volume reduction of thyroid nodules is already evident at 6 mo after RFA and results obtained early are stable over time.

## Discussion

Our study demonstrated the efficacy of RFA according to the types of benign thyroid nodules with a 1-year observation time. In this study, RFA effectively reduced nodular size by 90% approximately. It shows that RFA can be an effective way for the treatment of benign thyroid nodules^21^.

For MDRR and VRR, no significant differences were found after 12 months between the two groups, while the cystic-solid group achieved better results than the solid group at the 3rd and 6th month. This could be explained by the aspiration of cystic fluid and the transformation of nodular characteristic. Due to the aspiration of cystic fluid, the MDRR and VRR of cystic-solid nodules exhibited greater increase than those of the solid nodules. In addition, in light of the shift from cystic-solid nodules to solid nodules after ablation, the result showed that there was no significant difference between the two groups at the 12th month.

In our research, there is an inconsistency between CSRR and MDRR or VRR. For instance, the maximum diameter and volume of solid nodules decreased most significantly at the 12th month, while the cosmetic scores improved most significantly at the 6th month. We interpret this phenomenon as the difference between calculation and sensation. CSRR improved obviously at the 6th month in terms of appearance, but it does not represent the peak of nodule maximum diameter and volume reduction ratio (only judged by calculation). Of course, there are also consistent situation, such as cystic-solid nodules, the cosmetic improvement accords with the peak of the maximum diameter or volume reduction rate. This may also be ascribed to the artificial acceleration of size change after aspiration of cystic fluid, thus moving the VRR′ peak forward, in line with the CSRR. Throughout the full data, the achieved conclusion between benign thyroid nodules and cystic-solid nodules was similar. It may be related to the fact that most of the benign nodules found in our daily life are cystic-solid, while malignant nodules are usually solid and not suitable for ablation.

During the RFA procedure, we adopted the “moving-shot technique”. It is more flexible than “fixed-needle technique”, and the size of the ablation unit can be adjusted by changing the rate of tip movement^17^. The vital structures around the nodule can be protected by accelerating the movement speed and using a relatively small ablation unit, while the safe area in the center of the nodule can prolong the action time and adopt a larger ablation unit. For the position with rich blood flow, tissue necrosis can be made more thoroughly by increasing the power and slowing down the movement speed or even transitioning to fixed ablation, in order to ensure the safety and long-term effect of ablation. On the contrary, single point fixed-needle technique can only extend the ablation area by prolonging the action time, and the shape of ablation unit cannot be changed. As a result, the ablation is often incomplete. Incomplete ablation usually leads to inadequate shrinkage of nodules and pathological hyperplasia in the margin of the residue, which may be related to thermal ablation stimulation. The method of multi-point and multi-needle not only increases the risk of injecting more times, but also hardly achieve complete ablation, because there are many vital structures (laryngeal recurrent nerve, trachea, esophagus) around the thyroid gland. Moreover, it is dangerous for the critical tissue to prolong the action time of the fixed needle tip.

To date, RFA has gained widespread use as an alternative to surgery for solid malignancies including the liver, kidneys and lungs^22^. The first experience of image-guided RFA with evaluation of efficacy and safety of ultrasound guided percutaneous RFA of thyroid nodules has been reported and applied in 2006. During the procedure, a RF generator is used to produce voltage between an active electrode (applicator) and a reference electrode (grounding pad). The voltage is used to establish an oscillating electric field, which in turn induces frictional heating by causing electrons to collide with nearby molecules nearest to the applicator. Tissue heating to temperatures greater than 60 °C causes immediate coagulation necrosis. Nevertheless, it could lead to evaporation and carbide and make damages to the surrounding tissue if the temperature is higher than 100 °C.

RFA-associated complications have been reported, including voice change, skin burns, hematoma formation, vomiting, brachial plexus injury, nodule rupture, permanent hypothyroidism and transient hyperthyroidism^23,24^. Reported the incidence of voice change after RFA is higher for recurrent thyroid cancers (7.95%) than for benign thyroid nodules (0.94%), possibly because of the absence of a safety area around recurrent tumors^25^. Voice changes are likely due to recurrent laryngeal nerve dysfunction, which can be caused by thermal injury. Thermal injury to the recurrent laryngeal nerve may be prevented by using the hydrodissection with continuous fluid infusion^18^. Before RFA, the nodules adjacent to the "dangerous triangle" of the neck or the medial capsule of the thyroid gland should be injected with local anesthetic or normal saline to form a liquid isolation zone, which can effectively prevent the thermal injury to the nerve during the process. Part of transient hoarseness dates back from the blocking effect of local anesthetic on recurrent laryngeal nerve, which can be recovered within a few hours. For the superficial nodules close to the thyroid capsule, the heat can easily spread to the anterior cervical muscles and cause neck pain during ablation. This phenomenon can also be avoided by hydrodissection^18^. Although the incidence of complications of radiofrequency ablation is low, it should be paid attention to. Complications can be prevented or properly handled by deepening understanding and mastering skills.

Voice changes after EA (ethanol ablation, EA) are very rare. Although the exact incidence of voice changes after EA is unclear, it is lower than the incidence of voice changes after RFA. A possible mechanism for voice changes is the leakage of ethanol outside the thyroid gland, causing damage to the recurrent laryngeal nerve. As compared with RFA, EA has the benefits of reduced cost, pain and risk of developing nerve injury. However, results of the previous study comparing the efficacy of the two treatment modalities for hepatocellular carcinoma have suggested that RFA may be more effective in reducing the volume, because it can produce larger zone of tumor destruction than EA. Local tumor recurrence rates of 0–25% and 3.2–33% have been reported after RFA and EA for recurrent thyroid cancers, respectively^26^.

Both RFA and MWA were certified effective and safe in treating BTNs^27^. Liu et al.^28^ treated 474 benign thyroid nodules with MWA and observed a conspicuous reduction of 90% in volume. MWA^29^ was testified effective and less stressful compared with surgical resection. A study showed that the RFA group was superior to the MWA group at the 6th, 12th month and the last follow-up^16^. Decreasing the power output during the MWA procedure might increase the MDRR and VRR when treating BTNs. Teng et al.^30^ describes the use of low power (20 W) MWA in the treatment of 15 patients with 21 nodules diagnosed as primary papillary thyroid microcarcinoma with a mean VRR as 98.78 ± 5.61% at 3-year follow-up. No recurrent nodule was found during the follow-up period.

Several studies compared LA to RFA and showed that the nodular VRR for RFA is higher than that for LA^31^. While in favor of LA is a recent study performed by Pacella et al.^32^ where in 601 nodules, a significant nodule shrinkage was observed in the LA group, particularly in large nodules. No matter which method has more advantages, RFA and LA are all safe and effective measures for their low complication rates, high nodular VRRs and the preservation of thyroid function^33,34^. Zhou et al.^35^ conducted a retrospective study on solitary papillary thyroid carcinoma (36 received LA and 45 underwent surgery). No significant difference was found in the recurrence rates between the LA and surgical group (5.6% vs. 6.7%). LA can be considered as an alternative in the local control of papillary thyroid microcarcinoma.

HIFU, as a needle-free thermal ablation method, was first applied to clinic in 2010. The coagulative necrosis in targeted zone occurs through a focused high energy ultrasound beam. Recently, HIFU has been used to treat breast cancer and uterine leiomyoma. However, this technique is still in its infancy for the therapy of benign or malignant thyroid nodules. Compared with RFA and MWA, HIFU has lower incidence of infection and higher acceptance of patients, which also requires less anesthetic dose. Pierpaolo Trimboli et al.^36^ evaluated the efficacy and safety of HIFU in the treatment of benign solid nodules, the results showed that the average volume decreased by more than 40% within 1 year, and no complications were noted.

Compared with EA, MWA and LA, RFA is more widely used in clinic for its superiority in volume reduction. Some researchers pointed out that the operator’s experience is the key to therapeutic effect^37^. Moreover, another pilot study led by Pierpaolo Trimboli et al.^38^ found that the energy delivered per mL with RFA is the only technical parameter obviously correlated with VRR. At present time, RFA is recommended for the treatment of low-risk papillary thyroid microcarcinoma because so far there is some scientific evidences on its effectiveness and safety^39–41^.

Our study has several limitations. First, the vascular score was not evaluated and analyzed before and after RFA. Second, the main one was the short follow-up. Moreover, this study was a retrospective design and its performance at a single center. The acquisition of clinical practice needs a more careful and critical appraisal of the data^42^. A future prospective, longer follow-up study is necessary to confirm our preliminary results.

## Conclusion

In conclusion, RFA is an effective technique for patients with BTNs due to its significant shrinkage of nodular volumes and the apparent improvement of cosmetic concerns related to the nodules. The achieved MDRR and VRR in the cystic-solid nodule group were significantly better than those in the solid nodule group at the 3rd and 6th month.

## References

[1] Tang XY (2017). Evaluation of the safety and efficacy of radiofrequency ablation for treating benign thyroid nodules. J. Cancer.

[2] Rabuffi P (2019). Treatment of thyroid nodules with radiofrequency: A 1-year follow-up experience. J. Ultrasound.

[3] Che Y (2015). Treatment of benign thyroid nodules: Comparison of surgery with radiofrequency ablation. AJNR Am. J. Neuroradiol..

[4] De Bernardi IC (2014). Vascular and interventional radiology radiofrequency ablation of benign thyroid nodules and recurrent thyroid cancers: Literature review. Radiol. Med. (Torino).

[5] Magri F (2020). Laser photocoagulation therapy for thyroid nodules: Long-term outcome and predictors of efficacy. J. Endocrinol. Investig..

[6] Morelli F (2016). Microwave ablation for thyroid nodules: A new string to the bow for percutaneous treatments?. Gland Surg..

[7] Lang BHH, Woo YC, Chiu KW (2020). Identifying predictive factors for efficacy in high intensity focused ultrasound (HIFU) ablation of benign thyroid nodules—A retrospective analysis. Int. J. Hyperth..

[8] Happel, C., Korkusuz, H., Koch, D. A., Grünwald, F. & Kranert, W. T. Combination of ultrasound guided percutaneous microwave ablation and radioiodine therapy in benign thyroid diseases. A suitable method to reduce the 131I activity and hospitalization time? *Nuklearmedizin* **54**(3), 118–124 (2015). 10.3413/Nukmed-0674-14-06 25586901

[9] Mader A (2017). Minimally invasive local ablative therapies in combination with radioiodine therapy in benign thyroid disease: Preparation, feasibility and efficiency—Preliminary results. Int. J. Hyperth..

[10] Korkusuz Y (2017). Comparison of mono- and bipolar radiofrequency ablation in benign thyroid disease. World J. Surg..

[11] Korkusuz Y (2016). Bipolar radiofrequency ablation of benign symptomatic thyroid nodules: Initial experience with bipolar radiofrequency. Rofo.

[12] Deandrea M (2015). Efficacy and safety of radiofrequency ablation versus observation for nonfunctioning benign thyroid nodules: A randomized controlled international collaborative trial. Thyroid.

[13] Suh CH, Baek JH, Choi YJ, Lee JH (2016). Efficacy and safety of radiofrequency and ethanol ablation for treating locally recurrent thyroid cancer: A systematic review and meta-analysis. Thyroid.

[14] Morelli F (2018). Cooled tip radiofrequency ablation of benign thyroid nodules: Preliminary experience with two different devices. Gland Surg..

[15] Zeng, Z. *et al*. Efficacy of ultrasound-guided radiofrequency ablation of parathyroid hyperplasia: Single session vs. two-session for effect on hypocalcemia. *Sci. Rep*. **10**(1), 6206 (2020). 10.1038/s41598-020-63299-8 PMC7148367 32277134

[16] Cheng Z (2017). US-guided percutaneous radiofrequency versus microwave ablation for benign thyroid nodules: A prospective multicenter study. Sci. Rep..

[17] Park HS (2017). Thyroid radiofrequency ablation: Updates on innovative devices and techniques. Korean J. Radiol..

[18] Tang XY (2018). Risk assessment and hydrodissection technique for radiofrequency ablation of thyroid benign nodules. J. Cancer.

[19] Sung JY (2013). Single-session treatment of benign cystic thyroid nodules with ethanol versus radiofrequency ablation: A prospective randomized study. Radiology.

[20] Trimboli P (2020). Efficacy of thermal ablation in benign non-functioning solid thyroid nodule: A systematic review and meta-analysis. Endocrine.

[21] Pierre GDM, Nathalie R, Alban D, Arnaud H, Sykiotis GP (2019). Radiofrequency ablation of thyroid nodules: An alternative to surgery or first-line treatment?. Revue Medicale Suisse.

[22] Julie B (2015). Percutaneous hepatic radiofrequency for hepatocellular carcinoma: Results and outcome of 46 patients. Hepat. Med. Evid. Res..

[23] Aysan E, Idiz UO, Akbulut H, Elmas L (2016). Single-session radiofrequency ablation on benign thyroid nodules: A prospective single center study: Radiofrequency ablation on thyroid. Langenbecks Arch. Surg..

[24] Xu D (2016). Radiofrequency ablation for postsurgical thyroid removal of differentiated thyroid carcinoma. Am. J. Transl. Res..

[25] Chung SR (2017). Safety of radiofrequency ablation of benign thyroid nodules and recurrent thyroid cancers: A systematic review and meta-analysis. Int. J. Hyperth..

[26] Shin JE, Baek JH, Lee JH (2013). Radiofrequency and ethanol ablation for the treatment of recurrent thyroid cancers: Current status and challenges. Curr. Opin. Oncol..

[27] Hu K (2019). Comparison between ultrasound-guided percutaneous radiofrequency and microwave ablation in benign thyroid nodules. J. Cancer Res. Ther..

[28] Liu YJ, Qian LX, Liu D, Zhao JF (2017). Ultrasound-guided microwave ablation in the treatment of benign thyroid nodules in 435 patients. Exp. Biol. Med. (Maywood)..

[29] Liu SY (2019). Comparison of stress response following microwave ablation and surgical resection of benign thyroid nodules. Endocrine.

[30] Teng DK (2018). Long-term efficacy of ultrasound-guided low power microwave ablation for the treatment of primary papillary thyroid microcarcinoma: A 3-year follow-up study. J. Cancer Res. Clin. Oncol..

[31] Ha EJ (2015). Comparative efficacy of radiofrequency and laser ablation for the treatment of benign thyroid nodules: Systematic review including traditional pooling and Bayesian network meta-analysis. J. Clin. Endocrinol. Metab..

[32] Pacella CM (2017). A comparison of laser with radiofrequency ablation for the treatment of benign thyroid nodules: A propensity score matching analysis. Int. J. Hyperth..

[33] Papini E (2014). Long-term efficacy of ultrasound-guided laser ablation for benign solid thyroid nodules. Results of a three-year multicenter prospective randomized trial. J. Clin. Endocrinol. Metab..

[34] Ji HM (2015). Radiofrequency ablation is a thyroid function-preserving treatment for patients with bilateral benign thyroid nodules. J. Vasc. Interv. Radiol..

[35] Zhou W (2019). Ultrasound-guided laser ablation versus surgery for solitary papillary thyroid microcarcinoma: A retrospective study. Int. J. Hyperth..

[36] Trimboli P, Pelloni F, Bini F, Marinozzi F, Giovanella L (2019). High-intensity focused ultrasound (HIFU) for benign thyroid nodules: 2-year follow-up results. Endocrine.

[37] Bernardi S (2016). Radiofrequency ablation for benign thyroid nodules. J. Endocrinol. Investig..

[38] Trimboli P, Deandrea M (2020). Treating thyroid nodules by radiofrequency: Is the delivered energy correlated with the volume reduction rate? A pilot study. Endocrine.

[39] Lim HK (2019). US-guided radiofrequency ablation for low-risk papillary thyroid microcarcinoma: Efficacy and safety in a large population. Korean J. Radiol..

[40] Tong M (2019). Efficacy and safety of radiofrequency, microwave and laser ablation for treating papillary thyroid microcarcinoma: A systematic review and meta-analysis. Int. J. Hyperth..

[41] Xiao J (2020). Efficacy and safety of ultrasonography-guided radiofrequency ablation for the treatment of T1bN0M0 papillary thyroid carcinoma: A retrospective study. Int. J. Hyperth..

[42] Pacella CM, Papini E (2020). Thermal ablation procedures: The need of careful appraisal. Endocrine.

